# Society Transactions

**Published:** 1911-10

**Authors:** 


### BUFFALO MEDICAL JOURNAL

**A Monthly Review of Medicine and Surgery**

*EDITOR*

A. L. BENEDICT.

All communications, whether of a literary or business nature, books for review and exchanges, should be addressed to the editor, 354 Franklin Street, Buffalo, N. Y Please make personal or telephonic calls before 1 p. m.

The Buffalo Medical Journal will publish regularly, full transactions of any professional organization in western New York at cost. Personal notices, brief reports of society proceedings, snd the like, are always welcome and wiil be published without charge.

Subscriptions may commence at any time. $2.00 per year in advance,

Vol. LXVII. OCTOBER, 1911. No. 3

### Society Transactions

The Buffalo Medical Journal wishes to represent the interests of the medical profession of western New York generally and will gladly publish *brief* abstracts of proceedings of any society in its field. Papers embodying original research and observations of exceptional interest to the profession, will also be welcome. Any organisation desiring to publish its transactions in full may arrange for the printing of supplementary pages forming an integral part of the Journal at actual cost.

